# CAR-NK cells: harnessing the power of natural killers for advanced cancer therapy

**DOI:** 10.3389/fimmu.2025.1603757

**Published:** 2025-05-30

**Authors:** Filipa D. dos Reis, Yanis Saidani, Paula Martín-Rubio, Rebeca Sanz-Pamplona, Ana Stojanovic, Margareta P. Correia

**Affiliations:** ^1^ Cancer Biology and Epigenetics Group, Research Center of IPO Porto (CI-IPOP), CI-IPOP@RISE (Health Research Network), Portuguese Oncology Institute of Porto (IPO Porto), Porto Comprehensive Cancer Center Raquel Seruca (Porto.CCC), Porto, Portugal; ^2^ Doctoral Program in Biomedical Sciences, ICBAS - School of Medicine and Biomedical Sciences, University of Porto, Porto, Portugal; ^3^ Aix Marseille Univ, Centre National de la Recherche Scientifique (CNRS), Institut National de la Santé et de la Recherche Médicale (INSERM), Institut Paoli-Calmettes, Centre de Recherche en Cancérologie de Marseille (CRCM), Marseille, France; ^4^ Cancer Heterogeneity and Immunomics (CHI), University Hospital Lozano Blesa, Aragon Health Research Institute (IISA), Zaragoza, Spain; ^5^ Aragonese Foundation for Research and Development (ARAID), Zaragoza, Spain; ^6^ CIBERESP, Instituto de Salud Carlos III, Madrid, Spain; ^7^ Department of Immunobiochemistry, Medical Faculty Mannheim, Heidelberg University, Mannheim, Germany; ^8^ Mannheim Institute for Innate Immunoscience (MI3), Medical Faculty Mannheim, Heidelberg University, Mannheim, Germany; ^9^ Department of Pathology and Molecular Immunology, ICBAS - School of Medicine & Biomedical Sciences, University of Porto, Porto, Portugal

**Keywords:** CAR-NK cells, CAR (chimeric antigen receptor), adoptive cell immunotherapy, clinical trials, experimental models, metastasis, epigenetics, NK cells

## Abstract

Generation of Chimeric Antigen Receptors (CARs) presented a significant advance in the field of immunotherapy, allowing the targeting of cell-surface expressed molecules in an MHC-independent manner. Arming NK cells with CARs merges their innate natural cytotoxicity with the refined precision of targeted antigen recognition. The success of these therapies hinges on selecting the right tumor-specific targets to ensure effective activation and avoid self-reactivity. Optimization of CAR design and targeting is based on NK cell intrinsic properties (CAR modules and sources of NK cells), as well as on NK-tumor cell interactions (multi-antigen, multi-step, multi-switch). Additionally, the dynamics of tumor infiltration and adaptation to the tumor microenvironment play a critical role in CAR-NK cell efficacy. Combining CAR-NK cell therapies with chemotherapy, radiotherapy, checkpoint inhibitors, and emerging approaches like epigenetic modulators and oncolytic viruses, may address some of these challenges. The development of CAR-NK cell strategies for metastatic disease is especially promising, though the complexities of metastasis require refined targeted designs. As immunomics and multi-omics continue to evolve, the potential for designing more effective CAR-NK cell therapies expands. As results from preclinical and clinical trials unfold, a multidisciplinary approach integrating all those aspects will be key to unlock the full potential of CAR-NK cell-based adoptive transfers.

## 1 Introduction

Chimeric Antigen Receptor (CAR) immunotherapy has emerged as a promising approach for cancer treatment. In fact, having demonstrated substantial efficacy in pre-clinical and clinical studies, the U.S. Food and Drug Administration (FDA) has approved, to date, seven CAR-T cell therapies for the treatment of various hematologic cancers (1, 2). Despite this encouraging progress, CAR-T cell-based therapies face several challenges, including risk of severe side effects, such as cytokine release syndrome (CRS), neurotoxicity, and graft-versus-host disease (GvHD) (3–5). By combining CAR specificity with NK cell natural cytotoxicity, CAR-NK cells may tackle some of the challenges faced by CAR-T cell therapies. As part of the innate immune system, NK cells have the ability to target tumor cells in an antigen-independent manner, rapidly eliminating target cells through the release of perforins and granzymes. NK cell activity is regulated by the integrated balance of activating and inhibitory receptors that interact with specific ligands on the surface of tumor, virus-infected or transformed cells (6–8). This capacity allows for CAR-NK cells to combine CAR-dependent and -independent killing of tumor cells, particularly important to target tumor heterogeneity. Moreover, unlike CAR-T cells, CAR-NK cells can be derived from allogeneic donors, as they do not require Human Leukocyte Antigen (HLA) matching or prior antigen presentation, making them suitable for “off-the-shelf” use, substantially reducing production time and costs, and increasing scalability and accessibility (3, 5, 9–11). Early clinical data indicate that the safety profile of CAR-NK cell therapy holds an additional advantage, as the risk of CRS, GvHD and other severe adverse effects is lower and better manageable compared with CAR-T cells (5, 9). Comprehensive comparisons between CAR-NK and CAR-T cell therapies have been extensively described elsewhere previously (12, 13).

As the field evolves, CAR-NK cell-based therapies stand as promising approaches for the treatment of several cancer types, including solid tumors. However, employing CAR-NK cells to target solid tumors presents several obstacles inherent to their complexity. Solid malignancies exhibit physical barriers, such as dense extracellular matrix (ECM) and abnormal vasculature, restricting NK cell access (10, 13, 14). Strategies to increase CAR-NK cell infiltration are under development, including genetic engineering of chemokine receptors, such as CXCR1/2 and CXCR4, to enhance chemokine-guided migration (15, 16). Moreover, the tumor microenvironment (TME) can create immune hostile conditions through hypoxia, low pH, nutrient deprivation, and inhibitory factors like PD-L1, TGF-β, and adenosine, which can lead to NK cell impaired cytotoxicity and persistence. Approaches being explored include pharmacological inhibition of TGF-β and cytokine support (e.g. IL-15) to increase NK persistence and activation (13, 17). In addition, besides developing strategies to evade NK cell immune surveillance, such as downregulation of NK cell ligands, solid tumors typically display complex clonal heterogeneity and shared antigen expression between tumor and healthy tissues, increasing the complexity of applying CAR-NK cell therapies to target these malignancies (10). Nevertheless, several NK-CAR strategies are being developed and combined with other therapeutic approaches to overcome these hurdles, with multiple clinical trials on solid tumor currently underway.

In this review, we discuss methods for CAR-NK cell production, explore various preclinical tumor models, and provide an update on the current status of CAR-NK clinical trials. In addition, we delve into the promise of combining CAR-NK therapies with different therapeutic approaches, the relevance of this adoptive therapy to tackle metastatic disease, and the need of integrating the usage of computational biology to further potentiate CAR-NK cell therapy efficacy.

## 2 CAR-NK generation: sources and methodologies

### 2.1 Advances in CAR-NK cell constructs

Recent advances in CAR-NK cell constructs are driven by an increased understanding of NK cell biology and the desire to harness their unique activation mechanisms for cancer immunotherapy. The CAR construct itself typically comprises an extracellular antigen recognition domain, usually consisting of a single-chain variable fragment (scFv), a transmembrane domain, and intracellular signaling domains (4, 18). Different CAR generations are emerging, with increasing complexity on the composition of their intracellular signaling domains. While first-generation CARs contained only one signaling domain, second and third-generation CARs have been designed to further incorporate one or multiple co-stimulatory signaling endodomains, respectively (4, 10). These co-stimulatory signaling domains can include CD28, 4-1BB (CD137), OX40 (CD134) (11), 2B4 (CD244) and DNAX Accessory Molecule-1 (DNAM-1)/CD226 domains (19). Besides the conventional CD3ζ signaling domain, CAR-NK cells often incorporate intracellular signaling domains downstream of activating NK receptors, such as DNAX-activation protein 12 (DAP12) and DAP10 (20, 21). These signaling modifications aim to better align CAR-NK functionality with the innate cytotoxic pathways of NK cells.

To further improve the precision and efficacy of CAR-NK cell therapy, innovative “current-generation” CAR constructs have been designed (11). Bi-specific CARs are engineered to recognize two distinct tumor antigens simultaneously, reducing the risk of tumor escape due to antigen loss or inter- and intra-patient heterogeneity. For B-cell malignancies, bi-specific CARs targeting both CD19 and CD22 have shown promise in increasing tumor targeting and improving treatment outcomes in a murine lymphoma model (22). Moreover, advances in genetic engineering have enabled the development of cytokine armoring, intending to enhance CAR-NK cell persistence and function by providing autocrine cytokine support. This can be achieved through the release of soluble cytokines into the TME, such as IL-15, or by engineering cytokines in membrane-bound form, thus inducing immune responses upon cell-to-cell contact (11).

**Figure 1:**
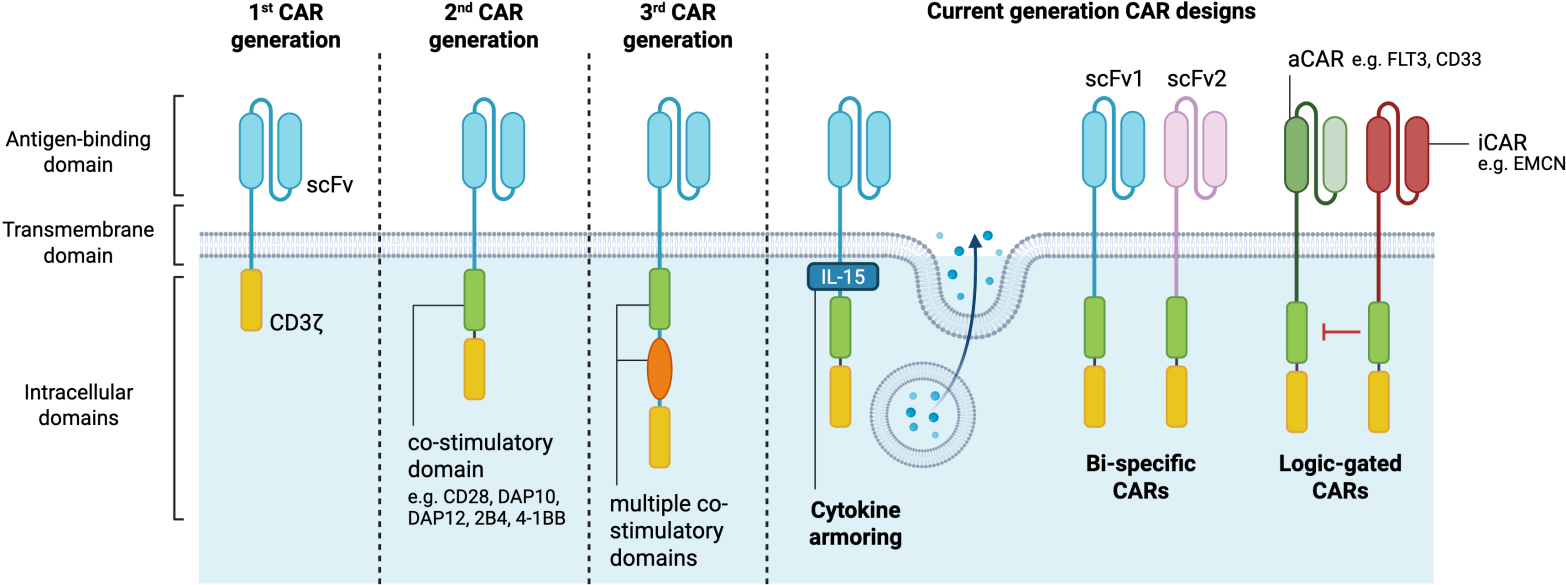
CAR-NK cell generations. The structural components of CARs comprise an extracellular antigen binding domain consisting of a single-chain variable fragment, a transmembrane domain and intracellular signaling domains. First CAR generations are composed of only one intracellular signaling domain, while second and third generations have additional co-stimulatory domains which potentiate the CAR-NK cell anti-tumor cytotoxicity. Current generation CAR designs allow for cytokine secretion, targeting of multiple antigens, and selective cell antigen targeting (logic-gated) to further improve the therapeutic activity of CAR-based immunotherapies. aCAR, activating chimeric antigen receptor; EMCN, endomucin; iCAR, inhibitory chimeric antigen receptor; scFV, single-chain variable fragment. Created in https://BioRender.com.

Another notable advancement is the development of logic-gated CARs that require multiple signals for activation. These CARs can reduce off-tumor toxicity by ensuring full NK cell activation only in the presence of a specific combination of tumor antigens. For instance, AND-gate CARs are activated only when two target antigens are present, while OR-gate CARs are triggered by either of two antigens (23). A notable example is the development of SENTI-202, an off-the-shelf CAR-NK cell therapy incorporating both “OR” and “NOT” logic gates. This construct enables CAR-NK cells to target acute myeloid leukemia (AML) cells expressing either FLT3 or CD33 (OR gate), while sparing healthy hematopoietic stem cells which express the endomucin (EMCN) (NOT gate). The FLT3-CD33 OR-gate CAR construct outperformed significantly single target CARs against FLT3 or CD33, both *in vitro* and *in vivo*. The FLT3-EMCN NOT-gate mediated a preferential killing of FLT3^+^ EMCN^-^ (AML-like), while preserving the double positive population (hematopoietic stem cell (HSC)-like) (24). SynNotch receptors represent an even more sophisticated approach, enabling multi-step tumor recognition. SynNotch receptors respond to an antigen by inducing expression of a CAR for another antigen, thus creating a sequential activation process that significantly enhances specificity (25). Finally, CAR-NK cells engineered to express CD19-CAR, IL-15 and inducible caspase 9, as a safety switch, have been administered to patients with relapsed or refractory CD19-positive cancers (17). The safety switch, which can be activated by rimiducid, was introduced to serve as a control measure to mitigate the potential adverse events in patients. Nevertheless, the safety switch was not activated in this study. Collectively, these advanced CAR designs aim to improve the safety, precision, and therapeutic potential of CAR-NK cell therapies (**Figure 1**).

As the field continues to evolve, ongoing research into CAR structure optimization and clinical testing is essential to ensure the development of highly effective next-generation CAR-NK cell therapies for cancer treatment.

### 2.2 CAR-NK cell transduction methods

Classical CAR-T cell generation approaches typically rely on viral-vector delivery of CAR constructs to ensure high transduction efficiency and stable long-term expression. The most widely used vectors for this purpose are lentivirus, retrovirus, and adeno-associated virus (AAV) vectors. NK cells are notoriously difficult to transduce due to their inherent resistance to genetic modifications, particularly through traditional viral vectors (26). However, significant progress has been made in recent years, improving both the efficiency and safety of transduction methods. These advances include the development of optimized viral vectors and non-viral methods.

#### 2.2.1 Viral vectors

Lentiviral vectors are a primary tool for CAR-NK engineering (27). However, lentiviral envelope pseudotyping plays a crucial role in optimizing transduction efficiency in primary NK cells. Traditionally, CAR-T cell lentiviruses are pseudotyped with vesicular stomatitis virus G (VSV-G). VSV-G lentiviruses bind to the low-density lipoprotein receptor (LDL-R) on the surface of T cells (28). However, NK cells exhibit a very low LDL-R expression, rendering the transduction with such vectors poorly efficient (29). Alternatively, the baboon envelope (BaEV) has been shown to induce higher transduction efficiency both in freshly-isolated and cultured NK cells (30). Retroviral vectors have also been explored as a transgene delivery method. For instance, alpharetroviral vectors have been shown to induce more than 60% transduction efficiency and a stable CAR expression for several weeks (31, 32). Finally, adenoviral vectors offer high transduction efficiency without genomic integration, making them an attractive option for transient CAR expression, though this may require repeated administration to maintain therapeutic efficacy. Altogether, the viral vectors are well established transgene transfer platforms. However, viral systems come with several drawbacks, including increased immunogenicity, potential insertional mutagenesis, limited insert size, high costs for good manufacturing practice (GMP)-grade viral production, and complex regulatory hurdles. These challenges associated with viral vectors encouraged the exploration of new transgene transfer venues.

#### 2.2.2 Non-viral transduction methods

Non-viral methods enable cell transduction without the risk of insertional mutagenesis or other genomic alterations. The Sleeping Beauty (SB) system is a synthetic transposon platform consisting of two components: a transposon, which carries the CAR gene and selectable marker flanked by inverted repeat sequences for genome insertion, and a transposase enzyme that mediates “cut-and-paste” integration at TA dinucleotide sites. Unlike viral vectors, it avoids hotspot integration, reducing the risk of insertional mutagenesis (32). The PiggyBac transposon system is another non-viral gene delivery platform increasingly used for stable integration of genetic material, such as CAR constructs, into NK cells. Originally derived from the cabbage looper moth, PiggyBac has gained significant attention in CAR-NK cell therapy due to its high transposition efficiency and the ability to carry large genetic payloads. Similarly to the SB, the PiggyBac operates by a cut-and-paste mechanism, but integrates at a TTAA site, thus enabling the system to carry large payloads (33). A potential application of the larger capacity of the PiggyBac would be the delivery of multiple CARs, logic-gated CARs and the manipulation of the balance of specific genes responsible for sustaining and potentiating CAR-NK cells (34). The use of lipid nanoparticles in various contexts has been a hot topic for the last couple of years given their low immunogenicity, the efficiency of mRNA transfer and protein expression in often exceeding 80% of positive cells (35). However, this strategy is transient, and scalability could be challenging. Electroporation is another widely used approach to produce CAR-NK cells, which is compatible with both freshly-isolated and cultured NK cells. Given the relatively low costs and transient expression of the transgene, it could potentially be useful in studies involving multiple dosing. Recently, CAR-NK cells against ROR1 have been generated by electroporation for the treatment of neuroblastoma. The product has shown efficacy in killing target cells *in vitro* and significantly prolonging survival in preclinical settings (36).

In sum, given the advances in CAR-NK construct design and transduction methods, we can expect the development of more effective CAR-NK strategies that will enhance scalability, safety, and clinical efficacy.

### 2.3 CAR-NK cell sources

The complex biology of NK cells, characterized by the lack of major histocompatibility complex (MHC) restriction and the complex balance of activating and inhibitory cues required for cell activation, prevent them from inducing GvHD (37). Therefore, most NK cell-based therapies prioritize allogeneic sources to circumvent the biological, logistical and economic burdens associated with autologous approaches (38). NK cells can be obtained from several sources, including peripheral blood, cord blood, immortalized cell lines and induced pluripotent stem cells (iPSCs). Each of these sources can produce clinically relevant cell doses, can be engineered to express CARs, and have shown efficacy both in preclinical models and in in-human studies. However, they present distinct advantages and challenges, and may exhibit differential transcriptional, phenotypic, and functional characteristics.

#### 2.3.1 NK-92 cells

NK-92 is an immortalized NK cell line derived from a patient with non-Hodgkin lymphoma (NHL) in 1992 (39). CAR-NK-92 cells were the first NK cell-based immunotherapy to receive Investigational New Drug approval by the FDA for clinical testing (40). Unlike primary NK cells, NK-92 cells exhibit a homogeneous phenotype, allowing for consistent and large-scale expansion *in vitro*, which is advantageous for adoptive cell therapy (41). Moreover, due to the low expression of Killer-Cell Immunoglobulin-Like Receptors (KIRs) on the cell surface, the cell line displays high cytotoxic activity which can be further enhanced by CARs (42). However, the cell line lacks the expression of CD16 on its surface, thus cannot execute antibody-dependent cell cytotoxicity (43). Finally, given the cancerous origin of the NK-92 cell line, the derived products are required to undergo irradiation, which limits both the persistence *in vivo* and long-term efficacy.

#### 2.3.2 Induced pluripotent stem cells (iPSCs)

iPSCs have garnered growing interest as a source for CAR-NK cells due to their self-renewal capacity and ability to differentiate into functional NK cells. Large-scale production of CAR-NK cells from iPSCs offers standardized manufacturing and batch-to-batch consistency, making them a promising option for off-the-shelf immunotherapies. However, the use of iPSCs comes with both technical and economic challenges. Differentiating iPSCs into fully functional NK cells is a time-consuming and complex process. Additionally, iPSCs may retain epigenetic memory from their tissue of origin, which can potentially influence their terminal function (44). While the manufacturing costs of iPSC-derived CAR-NK cells are substantially lower than those of autologous CAR-T cells, they remain significant (4).

#### 2.3.3 Primary NK cells

Allogeneic CAR-NK cell therapy can be achieved by harvesting primary NK cells from either cord blood (CB-NK) or peripheral blood (PB-NK). CB-NK cells offer advantage by containing a rich population of naïve NK cells, and are readily available through cord blood biobanks. CB-derived CAR-NK cells have been utilized in several clinical trials, most notably for treating CD19-positive lymphoid malignancies. However, some concerns have been raised regarding the lower cytotoxic potential of CB-derived NK cells, as well as limited persistence and overall phenotypical and functional immaturity (18, 29, 45). Alternatively, peripheral blood serves as another important source for CAR-NK cells. Peripheral blood is easily accessible in most clinical settings, and NK cell isolation is well established and minimally invasive. Compared with complex manufacturing processes like iPSC-derived CAR-NK cells, using primary NK cells significantly reduces costs. However, their scalability remains limited, making it challenging to obtain clinically relevant doses for large-scale treatments, and the natural inter-donor variability leads to heterogeneity of the final product (29).

## 3 CAR-NK cells and clinical trials

A growing number of CAR-NK clinical trials are being registered and conducted for various tumor types and patient populations. These trials are predominantly in early phases (Phase I and Phase I/II), focusing on evaluating the safety and efficacy of CAR-NK cells. Most trials target relapsed or refractory (R/R) hematological cancers, such as acute lymphoblastic leukemia (ALL), NHL, and AML, with CD19 and CD33 being the most common target antigens. A notable portion of these trials use NK cells derived from cord blood, reflecting the ease of genetic modification and scalability of this source. Several institutions are at the forefront of CAR-NK research. The M.D. Anderson Cancer Center, for instance, is leading multiple trials using CB-derived CAR-NK cells to target CD5, CD19, CD70, and TROP2 in R/R hematological cancers and solid tumors, such as ovarian and pancreatic cancer. Chinese institutions, including Zhejiang University and PersonGen BioTherapeutics, are actively testing CAR-NK therapies for various cancers, with a particular focus on AML and solid tumors. Trials using NK-92 cell lines and iPSCs are also in progress, highlighting a growing interest in off-the-shelf NK cell products for broader clinical application. In addition to hematological cancers, CAR-NK trials targeting solid tumors, such as colorectal cancer (CRC), ovarian and pancreatic cancers, are gaining traction. These trials include targeting of TROP2, CD70, MUC1, Claudin 18.2, as well as NKG2D ligands.

### 3.1 CAR-NK cell clinical trials: where do we stand?

Although most CAR-NK cell clinical trials are still ongoing, there are already published papers showing evidence of clinical success. In 2018, Tang and colleagues reported for the first time the results of the clinical administration of CAR-NK cells, testing the safety of CD33-CAR-NK-92 cells in three R/R AML patients (NCT02944162, **Table 1**). The authors demonstrated that this therapy could be safely applied in a series of three increasing doses without substantial adverse effects. However, no obvious clinical efficacy was observed. These results were associated with low *in vivo* survival of irradiated NK-92 cells upon infusion, thus requiring further optimization (46).

**Table 1:** CAR-NK cell clinical trials.

Target antigen	Study Start	Cancer type	NK cell source	Sponsor	Phase	Status	Trial ID	Location
CD5	2024	R/R hematological malignancies	Cord blood	M.D. Anderson Cancer Center	Phase I/II	Recruiting	NCT05110742	USA
		R/R NK and T-cell malignancies	Not disclosed	GC Cell Corporation	Phase I	Not yet recruiting	NCT06699771	Korea
CD19	2024	R/R B-NHL	Cord blood	Second Affiliated Hospital, School of Medicine, Zhejiang University	Phase I	Not yet recruiting	NCT06707259	China
		R/R B-cell malignancies		Second Affiliated Hospital, School of Medicine, Zhejiang University	Phase I	Not yet recruiting	NCT06464861	Unknown
		R/R NHL	NK-92	ImmunityBio	Phase I	Recruiting	NCT06334991	South Africa
		R/R NHL		ImmunityBio	Phase I	Recruiting	NCT05618925	USA
		R/R ALL	Not disclosed	Shahid Beheshti University of Medical Sciences	Phase I/II	Not yet recruiting	NCT06631040	Iran
		B-cell malignancies		The Second Hospital of Shandong University	Early Phase I	Not yet recruiting	NCT06596057	China
		R/R NHL		Shanghai Simnova Biotechnology	Phase I	Recruiting	NCT06206902	China
	2023	R/R B-NHL	Allogeneic (NS)	Changhai Hospital	Early Phase I	Unknown	NCT05673447	China
		ALL, CLL, B-cell lymphoma		Xuzhou Medical University	Early Phase I	Recruiting	NCT05739227	China
	2022	R/R B-NHL	Cord Blood	Second Affiliated Hospital, School of Medicine, Zhejiang University	Phase I	Enrolling by invitation	NCT05472558	China
		B-cell malignancies	Allogeneic (NS)	920th Hospital of Joint Logistics Support Force of People’s Liberation Army of China	Phase I/II	Unknown	NCT05654038	China
		R/R B-cell malignancies		Affiliated Hospital to Academy of Military Medical Sciences	Phase I	Unknown	NCT05645601	China
		R/R ALL	Not disclosed	Shanghai Simnova Biotechnology Co.,Ltd.	Phase I	Completed	NCT05563545	China
		B cell malignancies		Beijing Boren Hospital	Phase I	Unknown	NCT05410041	China
	2021	R/R hematological malignancies	Cord blood	Wuhan Union Hospital	Phase I	Unknown	NCT04796675	China
		R/R NHL		Takeda	Phase II	Active, Not recruiting	NCT05020015	USA
		R/R NHL, CLL or B-ALL	Allogeneic (NS)	Nkarta, Inc.	Phase I	Active, Not recruiting	NCT05020678	USA, Australia
		R/R B-NHL		Second Affiliated Hospital, School of Medicine, Zhejiang University	Phase I	Unknown	NCT04887012	China
		R/R ALL	Not disclosed	Zhejiang University	Phase I	Unknown	NCT05379647	China
	2020	R/R B-NHL	Not disclosed	Xinqiao Hospital of Chongqing	Early Phase I	Unknown	NCT04639739	China
		R/R B-cell lymphoma, CLL	iPSCs	Fate Therapeutics	Phase I	Terminated	NCT04245722	USA
	2019	Leukemia NHL	Allogeneic	Timmune Biotech	Early Phase I	Unknown	NCT03910842	China
		R/R B-cell lymphoma	Not disclosed	Allife Medical Science and Technology	Early Phase I	Unknown	NCT03690310	Unknown
	2017	ALL, CLL, NHL	Cord blood	M.D. Anderson Cancer Center	Phase I/II	Completed	NCT03056339	USA
	2016	Hematological malignancies	NK-92	PersonGen BioTherapeutics	Phase I/II	Unknown	NCT02892695	China
CD19/CD22	2019	R/R B-cell lymphoma	Not disclosed	Allife Medical Science and Technology	Early Phase I	Unknown	NCT03824964	Unknown
CD19/CD70	2023	R/R B-NHL	Cord blood	Tongji University School of Medicine	Phase I/II	Recruiting	NCT05842707	China
	2022	R/R B-NHL	Cord blood	School of Medicine, Zhejiang University	Phase I	Recruiting	NCT05667155	China
CD70	2024	TCL, AML	Cord blood	School of Medicine, Zhejiang University	Phase I	Not yet recruiting	NCT06696846	China
	2023	Renal cell carcinoma Mesothelioma Osteosarcoma	Cord Blood	M.D. Anderson Cancer Center	Phase I/II	Recruiting	NCT05703854	USA
	2022	R/R hematological malignancies	Cord blood	M.D. Anderson Cancer Center	Phase I/II	Recruiting	NCT05092451	USA
CLL1	2024	R/R AML	Allogeneic/Autologous	Shanghai General Hospital	Phase I	Recruiting	NCT06307054	China
	2023	AML	iPSCs	Zhejiang University	Phase I	Recruiting	NCT06027853	China
CLL1/CD33	2024	AML	iPSCs	Zhejiang University	Phase I	Recruiting	NCT06367673	China
	2023	R/R AML	iPSCs	Institute of Hematology & Blood Diseases Hospital, China	Phase I	Not yet recruiting	NCT05987696	China
	2020	AML	Not disclosed	Wuxi People’s Hospital	Early Phase I	Unknown	NCT05215015	China
CD33	2021	R/R AML	Not disclosed	Xinqiao Hospital of Chongqing	Phase I	Unknown	NCT05008575	China
	2016	R/R AML	NK-92	PersonGen BioTherapeutics	Phase I/II	Unknown	NCT02944162	China
CD33/FLT3	2024	AML, MDS	Allogeneic	Senti Biosciences	Phase I	Recruiting	NCT06325748	USA Australia
CD123	2024	R/R AML, BPDCN	Not disclosed	Chongqing Precision Biotech Co., Ltd	Phase I	Recruiting	NCT06690827	China
	2023	RR AML, BPDCN	Not disclosed	Chongqing Precision Biotech	Phase I/II	Recruiting	NCT06006403	China
	2022	R/R AML	Allogeneic	Affiliated Hospital to Academy of Military Medical Sciences	Early Phase I	Recruiting	NCT05574608	China
BCMA	2024	R/R MM	Not disclosed	Shahid Beheshti University of Medical Sciences	Phase I/II	Not yet recruiting	NCT06242249	Iran
	2023	R/​R MM PCL	Allogeneic	Hrain Biotechnology	Early Phase I	Recruiting	NCT06045091	China
	2022	R/R MM	Allogeneic	Shenzhen Pregene Biopharma	Early Phase I	Unknown	NCT05652530	China
	2021	R/R MM	Cord blood	Xinqiao Hospital of Chongqing	Early Phase I	Unknown	NCT05008536	China
		MM	iPSCs	Fate Therapeutics	Phase I	Active, Not recruiting	NCT05182073	USA
	2019	R/R MM	NK-92	Asclepius	Phase I/II	Unknown	NCT03940833	China
BCMA/​GPRC5D	2024	R/R MM	Allogenic	RenJi Hospital	NA	Not yet recruiting	NCT06594211	China
TROP2	2024	NSCLC	Allogeneic	Henan Cancer Hospital	Phase I/II	Not yet recruiting	NCT06454890	China
		CRC	Cord Blood	M.D. Anderson Cancer Center	Phase I	Recruiting	NCT06358430	USA
	2023	Ovarian and pancreatic cancer	Cord blood	M.D. Anderson Cancer Center	Phase I/II	Recruiting	NCT05922930	USA
		Solid tumors		M.D. Anderson Cancer Center	Phase I	Recruiting	NCT06066424	USA
NKG2DL	2024	Pancreatic cancer	Not disclosed	Zhejiang University	Early Phase I	Recruiting	NCT06478459	China
		Pancreatic cancer	Not disclosed	Zhejiang University	Early Phase I	Recruiting	NCT06503497	China
		R/R MM	Not disclosed	Changzhou No.2 People’s Hospital	Early Phase I	Not yet recruiting	NCT06379451	China
	2023	Ovarian cancer	Not disclosed	Hangzhou Cheetah Cell Therapeutics	NA	Recruiting	NCT05776355	China
		R/R AML	Not disclosed	Zhejiang University	NA	Recruiting	NCT05734898	China
	2021	Metastatic colorectal cancer	Not disclosed	Zhejiang University	Phase I	Recruiting	NCT05213195	China
	2021	R/R AML	Cord blood	Hangzhou Cheetah Cell Therapeutics	NA	Terminated	NCT05247957	China
	2020	R/R AML, MDS	Allogeneic	Nkarta	Phase I	Active, Not recruiting	NCT04623944	USA
	2018	Metastatic solid tumors	Allogeneic/Autologous	The Third Affiliated Hospital of Guangzhou Medical University	Phase I	Unknown	NCT03415100	China
MICA/B	2024	Gynecological cancers	iPSCs	Masonic Cancer Center, University of Minnesota	Phase I	Recruiting	NCT06342986	USA
Claudin 18.2	2024	Gastric cancer, pancreatic cancer	Cord blood	Zhejiang Provincial People’s Hospital	Phase I	Recruiting	NCT06464965	China
MUC1	2016	R/R solid tumors	Placental HSC-derived	PersonGen BioTherapeutics	Phase I/II	Unknown	NCT02839954	China
DLL3	2022	SCLC	Not disclosed	Tianjin Medical University Cancer Institute and Hospital	Phase I	Unknown	NCT05507593	China
PD-L1	2021	GEJ, HNSCC	NK-92	National Cancer Institute	Phase II	Recruiting	NCT04847466	USA
CD22	2019	R/R B-cell lymphoma	Not disclosed	Allife Medical Science and Technology	Early Phase I	Unknown	NCT03692767	Unknown
ROBO1	2019	Solid tumors	Not disclosed	Asclepius	Phase I/II	Unknown	NCT03940820	China
	2019	Malignant tumors	Not disclosed	Asclepius	Phase I/II	Unknown	NCT03931720	China
	2019	Pancreatic cancer	Not disclosed	Asclepius	Phase I/II	Unknown	NCT03941457	China
5T4	2021	Solid tumors	Not disclosed	Wuxi People’s Hospital	Early Phase I	Unknown	NCT05194709	China
GPC3	2024	HCC	Allogeneic	Shanghai General Hospital, Shanghai Jiao Tong University School of Medicine	Early Phase I	Recruiting	NCT06652243	China
Mesothelin	2019	Epithelial Ovarian Cancer	Not disclosed	Allife Medical Science and Technology	Early Phase I	Unknown	NCT03692637	Unknown
PSMA	2018	mCRPC	Not disclosed	Allife Medical Science and Technology	Early Phase I	Unknown	NCT03692663	China
CLDN6/GPC3/MSLN/AXL	2022	Advanced solid tumors	PBMCs	Hospital of Guangzhou Medical University	Phase I	Recruiting	NCT05410717	China
HER2	2017	Glioblastoma	NK-92	Johann Wolfgang Goethe University Hospital	Phase I	Active, Not recruiting	NCT03383978	Germany

ALL, acute lymphoid leukemia; AML, acute myeloid leukemia; BPCDN, blastic plasmacytoid dendritic cell neoplasm; B-NHL, B cell non-Hodgkin lymphoma; CLL, chronic lymphocytic leukemia; CRC, colorectal cancer; GEJ, gastroesophageal junction cancers; HNSCC, head and neck squamous cell carcinoma; mCRPC, metastatic castration-resistant prostate cancer; MDS, myelodysplastic syndrome; MM, multiple myeloma; NA, not applicable; NHL, non-Hodgkin lymphoma; NS, not specified; NSCLC, non small cell lung cancer; R/R, relapsed or refractory; SCLC, small cell lung cancer; TCL, T-cell lymphoma; PCL, plasma cell leukemia; HCC, hepatocellular carcinoma; HSC, hematopoietic stem cells.

In a study conducted at the M.D. Anderson Cancer Center, CB-derived NK cells engineered with a CD19-CAR, IL-15, and inducible caspase 9 (iCasp9), were administrated to CD19^+^ R/R NHL and chronic lymphocytic leukemia (CLL) patients (NCT03056339, **Table 1**). An interim analysis of the first 11 patients revealed promising results, considering the complete remission rate of 64%, without reporting any CRS, GvHD, or neurotoxicity events. Additionally, the infused CAR-NK cells showed long-term persistence for at least 12 months, suggested to be associated with the inclusion of IL-15 in the construct (17). This clinical trial has since then been completed, and the final results were published in 2024. Treatment responses were rapid at all dose levels. For the 37 patients who received lymphodepletion and CAR-NK cell infusion, the 1-year complete response rate was 29.7%. For patients with NHL, CLL and diffuse large B-cell lymphoma (DLBCL), the 1-year cumulative complete response rates were 83%, 50% and 29%, respectively (47). Most recently, the results of a terminated phase 1 clinical trial exploring FT596, an iPSC-derived CD19-CAR NK cell therapy for B-cell lymphoma patients, have also shown durable responses upon treatment (NCT04245722, **Table 1**) (48).

Focusing on solid tumors, NKG2DL-CAR NK cell treatment has been administrated to three metastatic CRC patients (NCT03415100, **Table 1**) without serious adverse effects. Upon receiving intraperitoneal CAR-NK cell infusion, two patients with malignant ascites experienced a decrease in tumor cells. The third patient was injected directly at the site of metastasis and showed complete metabolic response, highlighting the potential of CAR-NK cells for the treatment of solid tumors (49). Additionally, a case report evaluating treatment using ROBO1-CAR NK cells in a liver metastatic pancreatic ductal adenocarcinoma patient revealed safe administration with no substantial side effects (NCT03941457, **Table 1**). Moreover, the pancreatic lesion and liver metastasis were controlled within 5 months. Unfortunately, the patient passed away 3 months later due to multiple organ failure related to tumor progression (50). Lastly, the phase 1 CAR2BRAIN clinical trial (NCT03383978, **Table 1**) aimed to determine the safety and feasibility of HER2-CAR NK cells in glioblastoma patients. Out of the 9 patients treated at the time of publication, 5 experienced stable disease from 7 to 37 weeks, and 4 showed disease progression. Of note, in agreement with the results from the previous studies, the safety profile was favourable, with neither neurotoxic nor systemic side effects (51). Overall, these early findings demonstrate the potential of CAR-NK cell therapy for the treatment of several cancer types, expanding beyond the traditionally targeted hematological malignancies, thus offering promising therapeutic potential for hard-to-treat solid tumors. As the field progresses, results from these early-phase trials will be crucial in determining the viability of CAR-NK therapies as a treatment option.

## 4 CAR-NK cells and pre-clinical models

**Figure 2:**
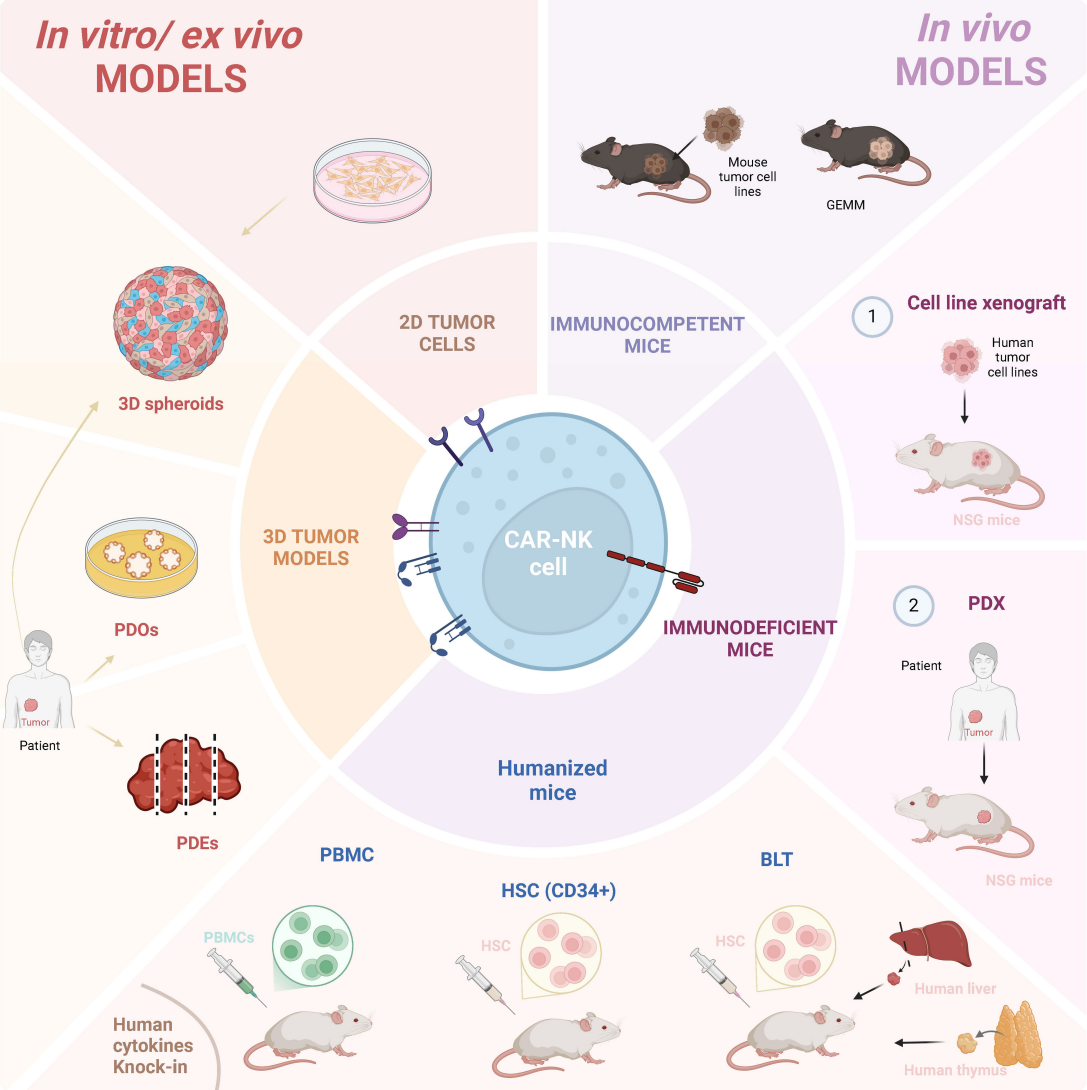
Immuno-models used for CAR-NK cell pre-clinical studies. CAR-NK cell pre-clinical research has been performed resourcing to several different models. *In vitro/ex-vivo* models include tumor cell lines and patient-derived material, in both 2D or 3D settings. 3D models can comprise spheroids, patient-derived organoids (PDOs) and patient-derived explants (PDEs). *In vivo* mouse models include immunocompetent mice inoculated with mouse tumor cells, or mice genetically-modified to generate tumors (GEMM). Immunodeficient mice, including NSG and humanized mice, can both be inoculated with (1) human tumor cell lines or (2) patient-derived explants. Humanized mice can be generated by inoculating human PBMCs or human hematopoietic stem cells (HSC). In addition, BLT (Bone Marrow-Liver-Thymus) mouse models are generated by the implantation of human liver and thymus tissues under the kidney capsule of immunodeficient mice, followed by the delivery of human HSCs, enabling the development of a functional human immune system. Created in https://BioRender.com.

The choice of pre-clinical models to test CAR-bearing cellular therapeutics depends on different factors, such as target antigen and tumor type, but also availability, scalability and price. A combination of both *in vitro* and *in vivo* models to comprehensively evaluate CAR-NK cell efficacy and safety before moving to clinical trials is frequently applied (**Figure 2**).

### 4.1 *In vitro/ex vivo* models

Initial testing of CAR-NK cell cytotoxicity against specific cancer cell lines is performed *in vitro* using both 2D and 3D based approaches. 3D models, such as tumor spheroids (52), can be used for high-throughput efficacy testing. Simple spheroids are typically derived from immortalized cancer cell lines, and fibroblasts, endothelial cells, or immune cells can be added to create more complex models that better replicate the TME. Within spheroids, tumor cells self-aggregate due to cell-cell adhesion and cell-matrix interactions, and frequently form layered zones of proliferating, quiescent, and necrotic cells. Although they can mimic topography and metabolic gradients of solid tumor tissue, they largely lack patient-specific relevance. There are, however, increasing efforts to utilize novel technologies, such as 3D bioprinting or microfluidic systems (53), to better represent complex structures of solid tumor tissue. Moreover, there are emerging spheroid tools based on cancer stem cells or fragmented tumor tissue, better representing patient heterogeneity and genetic traits of the original tumor (54). Spheroids are useful tools for proof-of-principle CAR-NK cell testing, as shown recently in the context of CAR-NK-92 cells secreting a peptide that blocks TGFβ1 signaling, which were tested using multicellular cancer spheroids and cancer-derived fibroblasts from patients with pancreatic cancer (55).

Alike spheroids, patient-derived organoids (PDOs) allow testing of CAR-NK cell migration and infiltration and can enable the evaluation of on-target/off-tumor effects using normal tissue organoids as controls. Tumor PDOs are re-constructed by cancer cells obtained from patient through biopsies, surgical resections, or from biological fluids (56, 57). They represent self-organizing cellular aggregates that mimic the architecture and the genomic landscape of the original tumor. Compared to spheroids, organoids allow differentiation into multiple cell lineages and therefore can have a multicellular identity that creates a more physiologically relevant model. However, as spheroids, they do not fully recapitulate the TME, as they miss vasculature, various tissue accessory cells, as well as tumor-infiltrating immune cells. PDOs can be used as an *in vitro/ex vivo* platform to evaluate the efficacy of CAR products in a personalized manner, as exemplified by Schnalzger et al. who generated luciferase‐expressing colorectal cancer organoids to quantify CAR-NK-92-mediated cytotoxicity (58). Although CAR-NK/NK92 products have been also tested against several tumor types, such as triple-negative breast cancer (59), pancreatic adenocarcinoma (60), esophageal cancer (61) and others, these studies are critically lacking a large number of patient-derived organoids to represent patient heterogeneity, urging the generation of biobanks available for testing and stratifying the patients for the best available treatment.

Patient-derived explants (PDEs) allow for *ex vivo* culture of freshly resected human tumor slices (62). Although limited in scalability, their advantage over PDOs is the retention of the original tumor architecture, microenvironment, and - importantly – the native infiltrating immune cell populations, enabling the evaluation of combination therapies that target other cells in TME. Due to short viability, PDEs can provide only immediate patient-proximal data. Unlike organoids, they cannot model tumor evolution over time or be used for genetic manipulation. However, they can be applied in combination with other approaches. By using malignant pleural mesothelioma explants treated with a STING agonist, Knelson and colleagues (63) identified activation of chemokine secretion by cancer cells and fibroblasts, along with differential T and NK cell resistance to treatment-induced cytotoxicity, which enabled downstream testing of mesothelin-specific CAR-NK cells in patient-derived organotypic tumor spheroids.

### 4.2 *In vivo* models

Pre-clinical mouse models are crucial tools for developing and evaluating cancer immunotherapies. They are successfully used to evaluate the efficacy of treatments, testing combinatorial therapies, and importantly, to study mechanisms of response and resistance. Currently, the available toolbox includes immunocompetent, humanized, and genetically engineered mouse models.

#### 4.2.1 Immunocompetent mice

Immunocompetent mouse models typically use syngeneic murine cancer cells that can be delivered via several routes of injection and implanted in various sites. Their main limitation is the kinetics of growth of the implanted tumor cells, whereby rapid tumor growth (avg. 2–4 weeks) is overemphasized (64). Mouse models mainly utilize pre-edited tumor cell lines lacking human-like immunoediting tumor evolution (elimination → equilibrium → escape) (65). In typical experiments, genetically homogeneous, inbred strains are used, failing to recapitulate human genetic diversity. While cancer risk increases exponentially with aging (66), the experiments are rarely performed in aged animals. The implanted tumors are often not orthotopic (grafted in the original organ site), and the tumor initiation (cell transformation, immune surveillance) is not recapitulated. Despite all this, mouse models do provide a physiologically relevant TME consistent of tumor cells, stromal cells and immune infiltrate that is shaped by the microenvironment. In the study of Look et al., murine CAR-NK cells were generated, and their efficacy was compared to CAR-T cells and CAR-macrophages in an orthotopic glioma model (67). The study indicated that all three CAR products succumbed to TME-mediated re-shaping, and that combination with cytokines benefited the outcome of the treatment. Mouse models can also be used to mechanistically address the actions of therapeutic agents in individual components of TME, e.g by applying targeted gene deletions (conditional gene knock-out models), and therefore address the complexity of cellular interactions in the context of tumor progression and response to therapy.

To more accurately reflect human cancer pathogenesis and allow for the study of tumor development and progression, genetically engineered mouse models (GEMMs) have been developed. Besides the assessment of therapy efficacy, they allow the exploration of carcinogenesis. In genetically engineered tumorigenic mouse models, tumors develop *de novo* in a natural immune-proficient microenvironment and closely mimic the histopathological features and genetic heterogeneity of their human counterparts. They are superior to cancer cell inoculation models, as they can recapitulate not only cancer heterogeneity, but also the metastatic cascade. In this regard, those models are primarily used to investigate NK cell “natural antitumor roles” and to highlight the mechanisms of suppression of their functions, therefore providing a basis for harnessing this knowledge in the context of immunotherapy.

#### 4.2.2 Immunodeficient and humanized mice models

Immunocompetent models are fully mouse systems and cannot be used to test human therapeutic products. Xenograft mouse models utilize immunodeficient mice co-engrafted with human tumors and human immune cells. The most commonly used immunodeficient mouse model is the NSG mouse strain (68–70) bearing IL-2 receptor gamma chain deficiency (IL2rγnull) on Non-Obese Diabetic (NOD)/severe combined immunodeficiency (SCID) background. These mice lack mature T, B, and NK cells, functional complement system, and display impaired macrophage and deficient dendritic activity.

The procedure utilizing NSG mice often involves low-grade irradiation that supports tumor cell engraftment. Frequently, tumor cells are delivered intravenously and subsequently form metastatic-like nodules in the lung. Tumor cells are often engineered to express luciferase, enabling their detection via measurement of bioluminescence. This allows follow-up measurements and assessment of therapy efficacy over time. In a recent publication, Li et al. used a preclinical xenograft model to show that tumor resistance to CAR-NK therapy is caused by a loss of metabolic fitness of NK cells after infusion in a lymphoma-bearing host (71). By applying single-cell transcriptomics, they demonstrated dynamic co-evolution of adoptively transferred NK cells and the tumor, whereby metabolically active tumor cells eventually progressed towards a NK-resistant phenotype. Although limited in scalability, they could show that in patients participating in a clinical trial utilizing their CAR-NK product (NCT03056339, see the **Table 1**), at single-cell level, NK cells bear similar traits as observed in preclinical model.

Although xenografts can recapitulate some aspects of the human immune-tumor interactions, they cannot evaluate the contribution of other cells to the therapy. Immunodeficient mice do not express human HLA molecules, lack a human endothelial barrier, and do not have fully developed lymph nodes with germinal centers that can participate in the immune reaction. In addition, adoptively transferred human immune cells into mice rely on endogenous growth factors, chemokines and cytokines, which often have low or no cross-reactivity, and cannot support their functions.

To establish a more human immune system in mice, several approaches have been developed (72, 73). Human peripheral blood mononuclear cells (PBMC)-humanized mice are created by injecting PBMCs into immunodeficient mice. Rapid engraftment of human immune cells can be achieved, albeit with differential efficacy for various immune cell types. However, these models allow only short-term studies due to GvHD development. Therefore, various approaches are undertaken to create next-generation models with reduced GvHD and improved engraftment, such as knock-out of MHC genes (74, 75). For example, in HUMAMICE, both murine MHC class I and MHC class II expression were eliminated, while simultaneously expressing human HLA-A2 (class I) and HLA-DR1 (class II) (76).

HSC-engrafted mice are created by injecting human CD34^+^ HSCs into immunodeficient mice (77, 78). They provide long-term engraftment, enable reconstitution of both innate and adaptive human immune cell populations and allow long-term studies. However, several cautionary factors should be considered when performing experiments in these animals. Donor variability can affect the engraftment rates and the differentiation of the different immune cell subpopulations, which in turn may impact the response to experimental treatments. NPI (NOD/SCID/IL2rγnull with human cytokine knock-ins) mice are advanced models engineered to express human cytokines on an NSG background, and can achieve engraftment with lower doses of human HSCs, showing improved development and function of human myeloid cells and NK cells (79, 80). Immune humanized mouse models are in the meantime commercially available and can be obtained as off-the-shelf ready-to-use products for immuno-oncology studies.

BLT (bone marrow-liver-thymus) humanized mice are a more advanced model, created by implanting human fetal liver and thymus tissue under the kidney capsule of immunodeficient mice, prior to human HSC injection (81–84). This procedure can be combined by provision of human cytokines using genetic knock-ins, as in NPI model. BLT mice develop a nearly complete human immune system, in which human T cells develop in the human thymus with proper HLA restriction. However, the procedure and the requirement of fetal tissues, which face ethical and regulatory challenges, limits the scalability of using these mice at large experimental testing.

Humanized mouse models can be used for the injection of patient cancer cells, or for the engraftment of PDEs, thus generating patient-derived xenografts (PDXs) (85). PDXs are increasingly used for drug development and precision oncology, as they provide personalized medicine approaches for individual patients. However, although they better reflect complexity of tumor biology and heterogeneity, and could aid expanding actionable genetic targets for treatment, they are not routine models used in academic research. In the context of CAR-NK therapy, humanized mouse nasopharyngeal carcinoma–patient-derived xenografts were used to show the efficacy of the combination therapy involving HSC–derived CAR-NK cells targeting programmed death-ligand 1 (PD-L1) and nivolumab (86). By comparing the PDX engrafted in NSG mice and humanized mice, this study also demonstrated that a humanized immune system differentially shaped the TME, more accurately mimicking the patient context.

Although humanized mouse models represent a significant breakthrough in immuno-oncology, they still face important limitations, particularly in recapitulating a fully functional human immune system due to species-specific differences in cytokine signaling, cellular interactions, and TME cues. Neverthless, although still limited in accurately predicting human immune responses and clinical outcomes, they remain a promising platform for pre-clinical evaluation, providing crucial insights that would be otherwise difficult to obtain *in vitro* or in human studies.

Optimal utilization of CAR-NK advanced tumor models requires collaboration across disciplines, including clinicians, researchers, and bioinformaticians, to standardize protocols, improve reproducibility, create centralized repositories, facilitate sharing of models and data, and to comprehensively analyze the data by leveraging advanced technologies (multi-omics, deep learning). While clinical studies performed so far show promise, at least for a subset of patients and some tumor types, a large load of groundwork is still required to understand complex mechanisms operating withing different tumors and individuals, and along trajectory of immune cell-tumor co-evolution, to improve current treatments and pave the road towards CAR-NK personalized therapy.

## 5 CAR-NK cell therapies and metastasis

Metastatic disease is responsible for about 90% of cancer-related deaths (87, 88). In recent decades, immunotherapy has emerged as a promising therapeutic strategy, but its success in the metastatic setting remains limited. Although metastatic spread is determined by intrinsic characteristics of tumor cells, the immune microenvironment also plays a crucial role in this process. Several studies highlight the important role of NK cells in controlling metastasis, from epithelial-mesenchymal transition (EMT) to the colonization of distant sites (89, 90). Thus, the anti-metastatic potential of NK cells has been extensively studied (91–93), and NK cell-based immunotherapies have been proposed as promising strategies against metastases. Among these approaches, CAR-NK cells represent a cutting-edge option due to their highly potentiated antitumor activity and scarce off-tumor toxicity. In the preclinical setting, some studies have reported the efficacy of CAR-NK cells as an encouraging therapy against metastases.

### 5.1 CAR-NK cells as a strategy against tumor metastasis

Different groups have studied CAR-NK cells as a putative strategy against breast cancer metastases. Mice bearing the human breast cancer cell line MDA-MB-453, treated with HER2-CAR-NK-92 cells, showed a significant decrease of tumor and lung metastasis formation compared to those receiving parental NK-92 cells (94). In another study, zEGFR-CAR-NK cells were implanted in a 3D-engineered hyaluronic acid (HA)-based niche for cell expansion. Mice treated with those cells showed a significant reduction in the number of lung metastases in an incompletely resected orthotopic breast cancer model (95). In a preclinical study, CAR-NK-92 cells targeting EGFR exhibited anti-tumor activity against breast cancer brain metastases, especially in combination with oncolytic herpes simplex virus 1 (96). Moreover, a biomimetic nanoplatform consisting of a combination of CAR-NK cell-derived exosomes (ExoCAR) and a nanobomb, produced a strong antitumor response *in vivo* against HER2-expressing breast cancer brain metastases, increasing mice survival (97). A next-generation CAR targeting CD44v6, a cell-surface glycoprotein implicated in tumorigenesis, tumor cell invasion and metastasis, has also been developed. CD44v6 CAR-NK cells demonstrated cytotoxicity against 3D spheroid models of triple-negative breast cancer (98).

Regarding lung metastases, CAR-NK-92 cells targeting ERbB2/HER2 have shown to reduce tumor growth in an experimental mouse model of lung metastases from renal carcinoma (99). Melanoma cell adhesion molecule (MCAM) is a relevant target, expressed on Ewing sarcoma (ES) and associated with metastasis. MCAM-CAR-NK cells significantly reduced lung metastasis and prolonged animal survival in an ES orthotopic xenograft mouse model (100). A different study used the ephrin type-A receptor-2 (EphA2) as a target antigen, showing that EphA2-CAR-NK-92 cells suppressed local tumor progression and metastatic burden in lungs in a sarcoma orthotopic mouse model (101).

Glypican-3 (GPC3)-specific CAR-NK-92 cells have been also explored, demonstrating potent antitumor effects both *in vitro* and *in vivo* against hepatocellular carcinoma (HCC). This CAR-NK product was also tested in an abdominal metastasis model, presenting better antitumor efficacy when injected intraperitoneally compared to intravenous administration (102).

The efficacy of adapter chimeric antigen receptor (AdCAR)-engineered NK-92 cells against bone metastases has also been demonstrated *in vitro*, in a panel of different cell lines derived from patient bone metastases, including those from mammary, renal cell and colorectal carcinoma, as well as melanoma (103). In differentiated thyroid cancer, it was observed that Thyroid-Stimulating Hormone Receptor (TSHR) is expressed not only in primary tumor, but also in metastatic sites. Accordingly, TSHR-CAR-NK-92 cells exhibited antigen-specific cytotoxic activity both *in vitro* and *in vivo* (104). Another targeted molecule, carcinoembryonic antigen (CEA), is a glycoprotein overexpressed in various epithelial tumors, including pancreatic, breast, lung, and colon cancer, associated with tumor differentiation, invasion, and metastasis. A next-generation CEA-CAR-NK-92 cells demonstrated effective cytotoxicity against colorectal cell lines and tumor spheroid models (105).

Finally, targeting cancer stem cells (CSCs) with CAR-engineered immune cells, including CAR-NK cells, is another promising strategy under investigation. CSCs represent a highly aggressive cell population responsible for metastases, tumor recurrence and chemoresistance, making them an interesting target to fight aggressive cancer (106–109).

### 5.2 CAR-NK cells and metastasis: clinical trials

In addition to the preclinical studies, some clinical trials have been conducted to test the efficacy of CAR-NK therapy in metastatic disease. In a clinical trial (NCT03941457, **Table 1**), a patient with pancreatic ductal adenocarcinoma and liver metastasis was treated with ROBO1-CAR-NK-92 cells, as described above. ROBO1 is a protein that plays a pivotal role in tumor angiogenesis and metastatic processes. The patient received CAR-NK treatment intravenously and by percutaneous administration to treat liver metastasis. No substantial treatment-related adverse events were reported. The patient had an 8-month overall survival, then dying due to tumor progression (50). Another clinical trial (NCT03415100, **Table 1**) explored CAR-NK cell therapy in three patients with metastatic CRC. In this case, the CAR construct was developed by fusing the extracellular domain of NKG2D to DAP12. Two patients were treated with intraperitoneal injection of low doses of the CAR-NK cells. These patients presented decreased ascites generation and a reduction in the number of tumor cells in ascites samples. The third patient, with liver metastases, was treated with intraperitoneal infusion of the CAR-NK cells, and achieved a rapid tumor regression in the liver after treatment (49). Finally, a phase II clinical trial (NCT04847466, **Table 1**) is evaluating the effectiveness of irradiated allogeneic PD-L1 CAR-NK cells in combination with pembrolizumab (PD-1 inhibitor) and N-803 (IL-15-based immunostimulatory fusion protein complex (IL15RaFc)), in patients with recurrent or metastatic gastric or head and neck cancer.

These findings highlight the growing potential of CAR-NK cells as a therapeutic strategy against metastatic disease, although further research is needed to fully understand their efficacy in clinical settings.

## 6 CAR-NK cells and combination therapies

**Figure 3:**
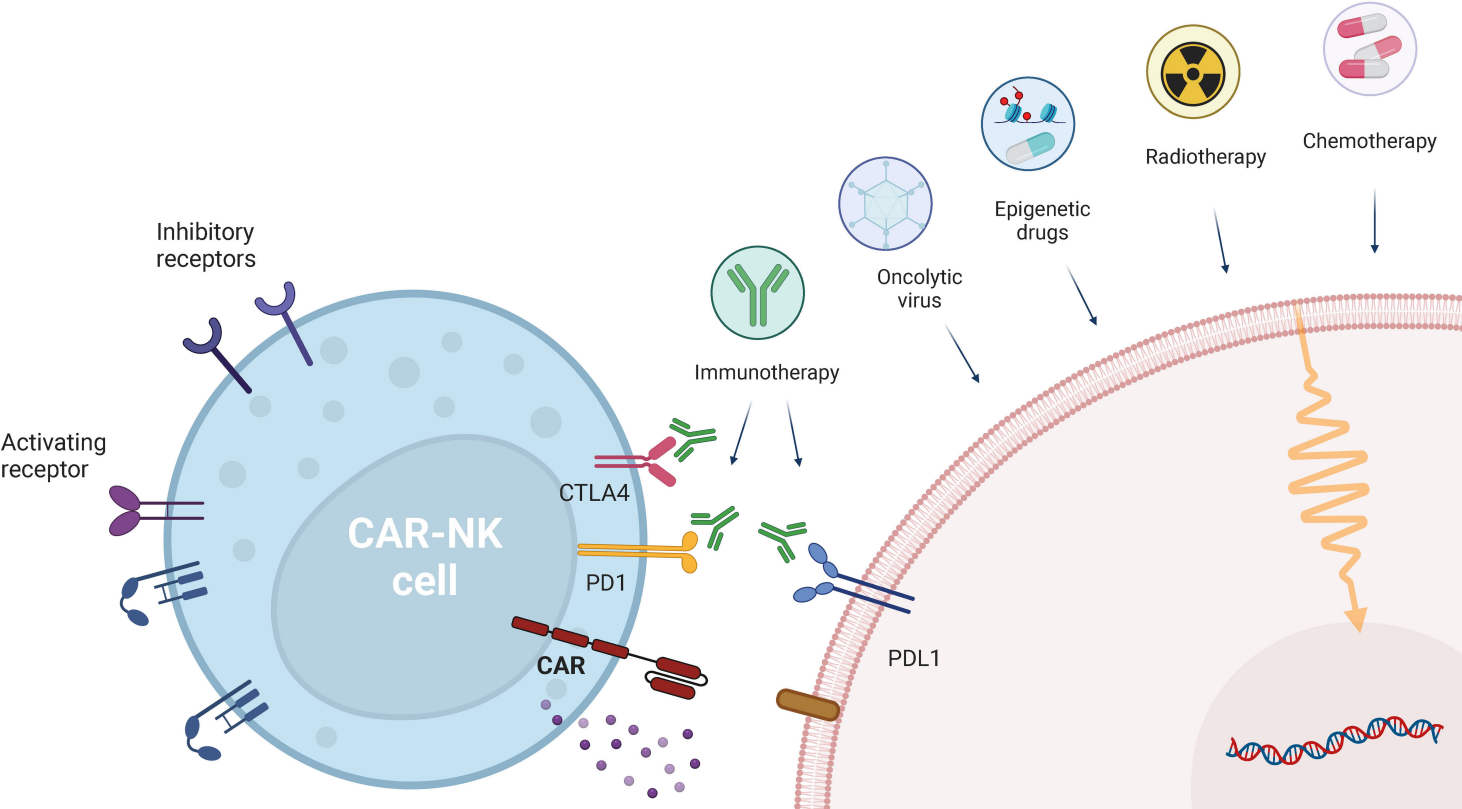
CAR-NK cell combination with other therapeutic approaches. CAR-NK therapies can be combined with other therapies, ranging from conventional therapeutic approaches (chemotherapy and radiotherapy), to checkpoint inhibition (anti-PD-1, anti-PDL-1 and anti-CTLA-4) and emerging therapies, such as epigenetic drugs and oncoviruses. Created in https://BioRender.com.

Combinatory therapies of CAR-NK cells with conventional therapeutic strategies, such as chemotherapy, radiotherapy, or immunotherapy, have emerged as promising strategies (**Figure 3**). Those approaches not only aim at overcoming challenges associated with CAR-NK cell-based therapies, but also to enhance their efficacy. By addressing key challenges, such as an immunosuppressive TME, poor tumor trafficking, reduced effector function, and downregulation of activating NK cell ligands and receptors, these approaches seek to break through critical barriers to effective treatment.

### 6.1 Immune checkpoint inhibitors (ICIs)

The programmed-cell death protein 1 (PD-1) immune checkpoint has been shown to be expressed on both circulating and tumor-infiltrating NK cells across several cancer types (110–115), being correlated with poor patient prognosis (116). Recent evidence also shows that this phenotype is associated with decreased NK cell activity, as demonstrated by impaired cytotoxicity, proliferation and cytokine production, which can be partially reverted through mAb-mediated disruption of PD-1/PD-L1 interactions (112, 113, 115–117). Following this rationale, combining checkpoint blockade with CAR-NK cell therapy could be a promising strategy to promote CAR-NK targeting of PD-L1-expressing tumors. Indeed, combination of CAR-NK-92 targeting prostate-specific membrane antigen (PSMA) with an anti-PD-L1 mAb (Atezolizumab) resulted in increased *in vitro* CAR-NK-92 mediated killing of C4-2 cells (a human castration-resistant prostate cancer (CRPC) cell line expressing PSMA) and primary tumor cells from a CRPC patient. Additionally, combination therapy enhanced *in vivo* cytotoxicity against C4–2 cells, leading to decreased tumor volume and longer survival in NOD/SCID mice, by directly blocking the PD-L1/PD-1 axis in both NK cells and CD8^+^ T cells (118). In another study, Liu et al. showed a synergistic antitumor response *in vivo*, upon treating a novel humanized nasopharyngeal carcinoma PDX mouse model with PD-L1 CAR-NK and an anti-PD-1 antibody (Nivolumab) (86).

The phase II clinical trial NCT04847466 (**Table 1**), referred above, is currently recruiting patients with recurrent/metastatic gastric or head and neck cancer, to test PD-L1 CAR-NK in combination with Pembrolizumab, together with an IL-15 superagonist. Additionally, in the CAR2BRAIN Phase I clinical trial (NCT03383978, **Table 1**), researchers are evaluating HER2-specific CAR-NK-92/5.28z cells, in combination with the anti-PD-1 mAb Ezabenlimab, for patients with recurrent HER2-positive glioblastoma (51, 119).

In addition, targeting NK inhibitory receptors have been reported to increase CAR-NK potential. A recent study showed that CD33-CAR-NK cells with CRISPR/Cas9-based disruption of the *KLRC1* gene, which encodes the NKG2A inhibitory receptor, exhibited increased cytotoxicity against AML cells, both *in vitro* and *in vivo* (120).

Targeting other immune checkpoints, such as cytotoxic T-lymphocyte associated protein 4 (CTLA-4), T cell immunoreceptor with immunoglobulin and ITIM domains (TIGIT), and T cell immunoglobulin and mucin-containing domain (Tim-)-3, in a CAR-NK combination context could also represent a strategy to increase CAR-NK cell cytotoxicity, since upon their inhibition, improved NK cell activity has been observed (121–126).

### 6.2 Radiotherapy

As a key approach in cancer treatment, radiotherapy directly causes tumor cell damage, leading to increased expression of antigens and cytokine release, ultimately impacting the TME (127, 128). Evidence suggests that radiation treatment increases NK cell infiltration and migration to the tumor site, while also modulating NK cell activity and tumor cell recognition. This effect is likely mediated by increased chemokine signaling and upregulation of activating ligands, such as MICA/B and ULBP-1, resulting in enhanced NK cell cytotoxicity (128–130). In 2024, a study by Lin et al. showed that pre-treatment of HCC cells with irradiation increased in vivo migration and activity of CXCR2-armed GPC3-targeting CAR-NK92 cells, through MICA/B and ULBP1 upregulation (131).

However, it is relevant to mention that radiation therapy has also been linked to a direct cytotoxic effect towards immune cells, including NK cells, leading to a decrease in circulating NK cell counts (128, 132). Although these findings highlight the potential synergy between radiotherapy and CAR-NK cells, the schedule modality of each approach should be taken into consideration.

### 6.3 Chemotherapy

Besides the standard lymphodepleting chemotherapy used before CAR-NK infusion, which aims to increase CAR-NK cell persistence and activity (119), conventional chemotherapy may also be used as an approach to improve CAR-NK cytotoxicity through direct tumor destruction, release of cytokines/chemokines and an overall decrease in immunosuppressive cell populations (133). In a recent study, treatment of primary ovarian cancer cells, harvested from ascites of an ovarian cancer patient, simultaneously using CD44-CAR-NK cells and cisplatin led to increased anti-tumor cytotoxicity, compared with monotherapies alone (134). Furthermore, combination of EGFR-CAR-NK-92 with cabozantinib showed a synergistic therapeutic effect against renal cell carcinoma (RCC) cell lines and in human RCC xenograft models, highlighting the potential of combining CAR-NK cells and chemotherapy as a promising strategy for the treatment of solid tumors (135).

### 6.4 Oncolytic viruses (OVs)

Oncolytic virotherapy relies on the use of viruses that replicate within cancer cells, directly leading to their death, while preserving normal cells and stimulating anti-tumor immune responses (136, 137). Thus, several studies have explored the possibility of combining OVs with CAR-NK cells. In an *in vivo* model of breast cancer brain metastases (BCBMs), intratumoral administration of oncolytic herpes simplex virus (oHSV) and EGFR-CAR-NK-92 cells resulted in improved killing of cancer cells and longer survival, when compared to monotherapy (96). Another study showed that treatment with herpes simplex 1-based OV expressing human IL-15/IL-15Rα sushi domain fusion protein (OV-IL15C), and EGFR CAR-NK cells, was able to synergistically suppress tumor growth in a glioblastoma mouse model, while also leading to increased survival and enhanced infiltration and activation of NK and CD8^+^ T cells (138). Evidence thus suggests that this combination approach may represent a powerful strategy to increase CAR-NK therapeutic success.

This approach may also be further extended to other viral-based platforms, such as virus-like particles (VLPs) and virus-mimicking nanoparticles (VMNs), which can stimulate the immune system and delivery of therapeutic cargoes (139), and may thus be considered to expand the CAR-NK combination toolbox.

Overall, exploiting the combination of CAR-NK cells with ICIs, radiotherapy, chemotherapy or OVs is an exciting opportunity to, not only overcome some of the challenges related with CAR-NK cell therapies, but also to further improve their efficacy by functioning as complementary strategies.

## 7 CAR-NK cells and epigenetics: what is in there?

Epigenetics involves heritable and reversible changes in gene expression that do not alter the DNA sequence itself. These changes comprise mechanisms such as DNA methylation, histone modifications, and microRNA regulation (140). DNA hypermethylation, driven by DNA methyltransferases (DNMTs) is known to lead to transcriptional repression. Histone proteins can undergo post-translational modifications, such as acetylation and methylation, which regulate chromatin structure. Histone acetylation, mediated by histone acetyltransferases (HATs), promotes gene transcription by creating an open chromatin state. In contrast, histone deacetylases (HDACs) remove acetyl groups, condensing chromatin and suppressing gene transcription. Histone methylation, catalyzed by histone methyltransferases (HMTs), involves the transfer of a methyl group to a lysine residue on a histone protein, either activating or repressing transcription, depending on the specific lysine residue and number of methyl groups added (140, 141).

Beyond regulating tumor development, recent research has highlighted the pivotal role of epigenetic mechanisms in shaping immune cell function and tumor cell recognition. Different studies have shown that treatment with epigenetic modulating drugs (epi-drugs), including DNMT, HDAC and HMT inhibitors, has led to increased expression of activating NK cell ligands, such as MICA/B, ULBP1-6, PVR and Nectin-2 (142–152). Additionally, NK cell function has also been shown to be modulated by epi-drug treatment. However, while some studies show increased expression of Perforin, CD107a, Granzyme-B/K, IFN-γ and TNF-α by NK cells (148, 153–157), leading to enhanced tumor cell killing, others report impaired degranulation and cytotoxic ability (152, 158–161). Thus, although promising, further studies are still necessary to fully depict the potential of epi-drugs as modulators of NK cell function. Recently, combination of CEA-CAR-NK-92 cells with the HDAC inhibitor sodium butyrate (NaB) led to CEA upregulation in CRC cells, increasing CEA-CAR-NK-92 cell-mediated killing in an *in vivo* model, leading to reduced tumor volumes (162). Moreover, in 2025, Tan et al. evaluated the combination of CEA-CAR-NK cells with STM2457, an inhibitor of METTL3, in an *in situ* CRC tumor immunocompetent mouse model (163). METTL3 is a writer of the epitranscriptomic m6A modification, which regulates splicing, stability and mRNA translation. Combination treatment showed significant tumor growth suppression, reduced CRC recurrence and increased NK cell infiltration within the TME (163). Notably, a multi-center Phase I clinical trial (NCT04623944, **Table 1**) is currently assessing allogeneic CAR-NK cells targeting NKG2D ligands, in the presence or absence of the DNMT inhibitor Decitabine, in patients with R/R AML and myelodysplastic syndrome (MDS).

Overall, these emerging findings highlight the potential of harnessing epigenetic mechanisms to potentiate CAR-NK cell therapies.

## 8 CAR-NK cell therapies: what can we learn from bioinformatics?

Nowadays, the analysis of omics data using bioinformatic techniques is becoming a standard practice in biomedical sciences. This approach enables a comprehensive understanding of the cell state, as it goes beyond the examination of a single gene, incorporating a broad spectrum of molecular data. Bioinformatics is a vast field. There exists a more specialized area called immunogenomics, consisting of the study of the immune system and the tumor-immune cells interactions. This bioinformatics approach can delve into the mechanisms of action of CAR-NK cells in two key areas: (i) the design of new CAR-NK cells, and (ii) the analysis of omics data derived from experiments involving CAR-NK cells.

As an example of CAR-NK design, Lee and colleagues used a data-driven bioinformatics approach to predict optimal antigen combinations of AML and healthy tissue target antigens to incorporate into CAR gene circuits based on OR or NOT logic gated CAR gene circuits. By using this pipeline, they designed the first-in-class CD33 OR FLT3 NOT EMCN gene circuit, as described above (24, 164). Peng et al. also used bioinformatic tools to address the expression and the prognostic role of c-Met as a target prior to developing c-Met-CAR-NK-92 cells specific for lung adenocarcinoma (165).

To understand CAR-NK mechanisms of action using immunogenomics, not only bioinformatics, but also artificial intelligence (AI) tools can process vast amounts of genomic and multi-omics data to identify biomarkers associated with immunotherapy responses (166). AI can assist in providing insights into the intricate molecular networks between the immune and the cancer cells. Several studies utilize omics data analysis, to evaluate functionality of CAR NK cells, particularly in preclinical studies. A common approach in these studies is the use of sequencing protein-coding transcriptome (RNAseq) (47, 167–169). For example, Silvestre et al. used RNAseq to evaluate transcriptomic profiles of CD19-CAR-NK-92 cells after coculture with target B cell lines. Their study revealed that fourth generation CAR.19-IL15/IL15Rα had improved proliferation and effectiveness compared with other NK-92 cell-based tumor therapies (169). Biggi and colleagues used RNAseq to assess differences between CAR.19 and CAR.19-IL-27 cells, and digital droplet PCR to study persistence of CAR NK cells in mouse blood during treatment (170). In the last years, single-cell sequencing is also being implemented to study CAR-NK cells in the preclinical settings (71, 171, 172). Namely, Li et al. conducted single-cell RNAseq to analyze the evolution of CAR-NK cells after adoptive transfer using an *in vivo* preclinical model and samples from patients responding and non-responding to CAR/IL15 NK cells (71).

Although immunomics approaches strongly supports personalized treatments with CAR-NK, some challenges remain, such as data quality, model interpretability, integration of multi-modal data, and privacy protection.

## 9 CAR-NK cells: where can we go?

CAR-NK cell knowledge is emerging, with a growing amount of translational research and early clinical trials substantiating their therapeutic potential. While several clinical trials are currently underway, only very few have been completed or have published results. Thus, the coming years will provide crucial insights demonstrating the true potential of CAR-NK strategies, and guide more refined and effective designs. CARs are designed to recognize tumor-specific surface molecules, a process that provides cell activation while ensuring no reactivity to self. Therefore, selecting the right target is vital. The CAR construct is comprised of modules that are designed for optimal cell activation by delivering co-stimulatory and pro-survival signals. In this regard, the design of optimal constructs for each CAR-NK cell product should take into consideration their origin, differentiation or haplotype, requiring further groundwork. Moreover, the spatiotemporal component is often underestimated in most of preclinical tests. CAR-NK cells need to reach and infiltrate tumor tissue, adapt to TME, and co-evolve with the tumor. These dynamics can affect CARs directly (eg. recognition, signaling), but also other regulatory hubs of NK cells, such as transcription, epigenome or protein synthesis. The combination of CAR-NK immunotherapy with other strategies, such as chemotherapy, radiotherapy, or checkpoint inhibitors, aims to address and overcome some of these issues. Moreover, emerging approaches, such as epigenetic modulating drugs and oncolytic viruses, hold promise and should be further explored. As NK cells are key effectors in controlling metastases, the development of CAR-NK strategies to tackle metastatic disease assumes particular relevance. However, the metastatic cascade is complex, and outgrowing metastases often show phenotypes and targets distinct from the primary tumor, which should further guide the design of CAR-NK strategies against metastatic disease. With the rising standing of immunomics for designing novel targets and innovative strategies, alongside the growing field of multi-omics that allows spatiotemporal mapping and identification of novel mechanisms operating at immune-tumor axis, the doors of opportunities to better design CAR-NK-based therapies, are wide open. Thus, it is crucial to pursue multidisciplinary approaches and to integrate computational biology into the study framework and in-depth analysis of pre-clinical and clinical results. NK-CAR therapies offer several advantages over CAR-T cells, including the possibility to use them “off-the-shelf”, a lower risk of GvHD and of severe CRS, and intrinsic anti-tumor activity that can target cancer cells through both CAR-dependent and CAR-independent mechanisms. Nevertheless, NK cells tend to persist for a shorter period, showing limited proliferation post-infusion, often requiring multiple administrations. Moreover, they are harder to genetically modify, displaying lower transduction/transfection efficiencies and lack classical memory (although they can exhibit ‘trained immunity’), which may make them less effective for long-term tumor control. Although several efforts have been made to overcome these hurdles, such as more effective transduction strategies and CAR designs incorporating cytokines to enhance their persistence, further refinement of NK-CAR strategies will be crucial to maximize their therapeutic potential. Moreover, recent findings showed that combination of CAR-T cells with a small number of CB-derived CAR-NK cells could significantly enhance therapy efficacy, particularly in the early phases of treatment. Bachiller and colleagues showed that CAR-T/CAR-NK co-administration appears to support early activation and migration of CAR-T cells toward multiple myeloma (MM) targets, improving cytotoxicity and decreasing T cell exhaustion, without increasing the risk of CRS. These results highlight a novel synergistic immunotherapeutic approach that may overcome some of the current limitations of each therapy (173).

CAR-NK cells represent a revolutionary advancement in cancer therapy. As basic, translational, and clinical research advances, the near future will provide clearer guidance on the trajectory of CAR-NK therapies toward their development as standard treatment strategies.
